# Extensively drug-resistant shigella with chlamydia coinfection in a person living with HIV: A case report

**DOI:** 10.1016/j.idcr.2026.e02826

**Published:** 2026-11-03

**Authors:** Marine Khachatryan, Nazar Akhverdyan, David Del Bello, Paul Nieberg

**Affiliations:** a Huntington Health, 100 W. California Blvd, Pasadena, CA, USA; b Division of Infectious Diseases, Huntington Health, 100 W. California Blvd, Pasadena, CA, USA

**Keywords:** Shigella, Chlamydia, Extensively drug-resistant, XDR Shigella, coinfection

## Abstract

Cases of drug-resistant *Shigella* are being reported across the United States. While traditionally linked to contaminated food and water, there is a rise in sexual transmission, especially among high-risk populations. We present a case of extensively drug-resistant *Shigella flexneri* and *Chlamydia trachomatis* co-infection in a person living with HIV.

## Highlights

• *Shigella* has emerged as a sexually transmitted infection.
• Lack of clinical response may indicate extensively drug-resistant *Shigella flexneri.*
• Co-infection with other sexually transmitted diseases is also emerging.
• Consider early initiation of broad-spectrum antibiotics in high-risk populations.

## Introduction

*Shigella* spp. are non-motile, gram-negative rods transmitted by direct and indirect fecal-oral contact. It is highly transmissible given the low inoculum needed to cause infection and short incubation period of approximately 1–3 days [1], [2]. Clinical manifestations include abdominal pain, diarrhea ranging from watery to bloody, vomiting, tenesmus, and fever. Complications include intestinal obstruction, colonic perforation, bacteremia, reactive arthritis, and hemolytic uremic syndrome [3]. While shigellosis can be self-limiting in immunocompetent individuals, antimicrobial therapy may reduce complications and duration of illness in immunocompromised individuals, such as people living with HIV (PLWHIV), in whom severe disease is more likely [4].

While traditionally associated with contaminated food and water, shigellosis has recently emerged as a sexually transmitted enteric infection, particularly among men who have sex with men (MSM) and PLWHIV. HIV, and specifically high viral load, has been shown to be an independent risk factor for shigellosis [5]. In a population-based study from Taiwan, high viral load, and other sexually transmitted infections (STIs), such as gonorrhea and syphilis, were also found to be independent risk factors for *Shigella* infection [5]. A study by Hughes *et al.* in the United Kingdom found that the prevalence of gastrointestinal infections in a sample of MSM diagnosed with chlamydia was 8.6%, with 1.8% testing positive for *Shigella* [6]. Finally, an analysis of 24 cases of *Shigella flexneri* in MSM showed that 40% had concomitant STIs of other etiologies [7]. Sexual transmission of *Shigella* has been associated with behaviors similar to those of other STIs such as condomless sexual intercourse, direct oral-anal contact, multiple sexual partners, and shared sexual networks [8]. Thus, co-infection with other STIs, such as *Chlamydia trachomatis*, should be considered given their shared transmission routes.

This is further complicated by the rise of extensively drug-resistant (XDR) *Shigella* in the United States, increased from 0% to 5% between 2015 and 2022, respectively [9]. These strains are resistant to first-line agents such as azithromycin, ciprofloxacin, ceftriaxone, trimethoprim-sulfamethoxazole, and ampicillin. Clinical suspicion for XDR strains should be heightened when these treatments do not improve symptoms. Of note, one possible driver of XDR *Shigella* strains in MSM may be related to repeated antimicrobial treatment of other STIs such as gonorrhea and chlamydia*,* which creates selective pressure [10]. Empiric broad-spectrum antibiotics such as carbapenems may be warranted while awaiting stool culture and susceptibility testing when there is concern for drug-resistance.

## Case presentation

A 41-year-old MSM, with a past medical history of HIV on Biktarvy (bictegravir, emtricitabine, tenofovir alafenamide), schizoaffective disorder, methamphetamine use, housing insecurity, heart failure with reduced ejection, and hypertension presented to the emergency department with 3 days of frequent non-bloody diarrhea, abdominal pain, nausea, and vomiting. Review of systems was negative for fever, chills, sore throat, urinary symptoms, melena, hematochezia, sick contacts, or recent travel.

Vital signs on admission included an elevated temperature of 100.6 °F, low-normal blood pressure of 93/73 mmHg, elevated heart rate of 129 beats per minute, and normal oxygen saturation of 98% on room air. Physical exam was notable for diffuse abdominal tenderness to palpation without guarding, rebound, or rigidity. Laboratory studies revealed a normal white blood cell count of 8.6 × 10^3^mL with 78.5% neutrophils, procalcitonin of 0.35 ng/mL, erythrocyte sedimentation rate of 7 mm/hour, and elevated C-reactive protein of 50 mg/dL. HIV studies showed an absolute CD4 count of 336 cells/mm^3^ and viral load of 5566 copy/mL. *Clostridioides difficile* polymerase chain reaction (PCR) was negative. Gastrointestinal PCR panel was positive for *Shigella* spp*.,* and stool culture later confirmed the presence of *Shigella flexneri*. Rectal and pharyngeal *Chlamydia trachomatis* PCR were also positive. Blood cultures were negative. Computed tomography of the abdomen and pelvis with contrast showed mild diffuse colonic wall thickening concerning for colitis.

The patient was started on intravenous ceftriaxone. Due to lack of clinical improvement and concern for antimicrobial resistance, therapy was broadened to meropenem on day 4 of hospitalization. Antimicrobial susceptibility testing later revealed extensively drug resistant *Shigella flexneri*, susceptible only to meropenem (minimum inhibitory concentration [MIC] < 0.5) and eravacycline (MIC 0.06) (Table 1). Susceptibility results were interpreted using Clinical and Laboratory Standards Institute (CLSI) M100 (35th edition, 2025) clinical breakpoints [11]. Meropenem was continued for an additional four days. He was also treated with doxycycline for rectal and pharyngeal *Chlamydia trachomatis* coinfection. The patient ultimately left against medical advice.

**Table 1:** Susceptibilities for *Shigella flexneri*.

Antibiotic	Minimum Inhibitory Concentration (mg/mL) [11]	Interpretation
Ampicillin	≥ 32	Resistant
Ceftriaxone	≥ 64	Resistant
Ciprofloxacin	≥ 4	Resistant
Meropenem	≤ 0.5	Susceptible
Trimethoprim/Sulfamethoxazole	≥ 4/76	Resistant
Doxycycline	≥ 16	Resistant
Tigecycline	0.06	None
Azithromycin	64	Resistant
Eravacycline	0.06	Susceptible

## Discussion

In conclusion, the incidence of shigellosis in the United States has risen over the last several years, as well as concern for co-infection with other STIs. This case report illustrates one such example of co-infection of *Shigella flexneri* and *Chlamydia trachomatis*, one which is not commonly described in the current literature. It highlights the importance of considering infection with enteric pathogens as being sexually transmitted, especially in certain populations such as MSM or patients with HIV. The detection of rectal and pharyngeal *Chlamydia trachomatis* in our patient strengthens the likelihood of sexual acquisition. Co-infection with other STIs, through overlapping social and sexual practices, highlights the need to pursue further STI testing in the right clinical setting [8]. Failure to perform STI testing may result in missed diagnoses and ongoing transmission. Additionally, clinicians should consider XDR *Shigella* in patients whose symptoms are not improving with traditional therapies such as azithromycin or ceftriaxone. MSM and PLWHIV are at higher risk of morbidity and mortality from the complications of fulminant disease. In these circumstances, one should consider starting carbapenem therapy while awaiting stool culture and susceptibilities – a microbiological study that may be overlooked in an era where PCR tests are becoming increasingly available.

## CRediT authorship contribution statement

**Marine Khachatryan:** Writing – review & editing, Writing – original draft, Investigation, Conceptualization. **Nazar Akhverdyan:** Writing – review & editing, Writing – original draft, Investigation. **David Del Bello:** Writing – review & editing, Supervision, Conceptualization. **Paul Nieberg:** Writing – review & editing, Supervision.

## Ethical approval

Not applicable.

## Consent

Consent to publish was not obtained for this case report as it does not contain any personal identifiers.

## Funding

The research did not receive any specific grant from funding agencies in the public, commercial or not-for-profit sectors.

## Declaration of Competing Interest

The authors have no conflicts of interest.

## Data Availability

Data sharing is not applicable to this article as no datasets were generated or analyzed during the current study.
